# A Sufficient Reply to the Anonymous Author of the Review of "a Treatise on the Phenomena of the Animal Magnetism, by Dr. Loewe,"

**Published:** 1823-01

**Authors:** M. Loewe

**Affiliations:** London


[The following paper from Dr. Loewe is the first protest we have received against any of the articles inserted in this Journal during our *censorship:* we have not suffered it to encroach upon the usual contents of the number, but have added an additional half sheet, for the express purpose of giving it a place, without depriving our readers of any more interesting matter. The account of Dr. Loewe's book was not a "review," as he states in his answer, but a sketch for our bibliographical department, transmitted to us by a gentleman whose residence in Berlin, and acquaintance with the different medical schools in Germany, enabled him to judge both of the individual merits of the book in question, and of its meagreness compared with other works on the same subject published in that country. Dr. Loewe entitles his "a *sufficient* Reply," &c., and our correspondent agreeing with the author that it is *quite sufficient*, our readers need not fear a continuation of the subject, as it is not our intention to defend the individual attacked, nor attempt to answer an effusion in which we cannot select any one passage as more objectionable than another.]

---

*A sufficient Reply to the anonymous Author of the Review of "A Treatise on the Phenomena of the Animal Magnetism, by* Dr. Loewe," *which appeared in the London Medical and Physical Journal for November*, 1822, *p.* 441, *&c. Art.* 2.

" Stultum est alios reprehendere, quorum virtutes ignoramus.

I should scarcely conceive it necessary to make any reply to the sarcastic composition of the Reviewer of my little Treatise, as such a method of reviewing a work is at once a proof, that the materials of which it is composed are far too high for the shallow comprehension of this pretended critic, and, at the same time, the highest commendation he could possibly bestow on it; but, to convince him how ridiculous he makes himself appear, and how illogically and illiberally he has written on the subject, I feel myself bound to shew publicly that his motive for writing was not a love for scientific truth, but merely to display, *as he conceived*, his own superior knowledge at the expense of another, whose abilities he probably considered too humble properly to handle so sublime a subject.

In the first place I observe, that this *learned* Critic has misrepresented the title of my little work: instead of "A Treatise on the *Phenomena* of Animal Magnetism," he calls it "A Treatise on *Animal Magnetism.*" Every one must know there is a wide difference between "A Treatise on the *Phenomena* of Animal Magnetism," and "A Treatise on *Animal Magnetism.*" For what purpose then, I would enquire of this *learned* Reviewer, did he thus misrepresent the title of my work? The same question will apply to that part of the review in which he introduces me to his readers as "*a* Doctor of Philosophy," &c. &c.; but, disdaining in the slightest degree to imitate his illiberality, I must be allowed to say, that he here loses all claim to the character of a Reviewer, and appears before the public as an ignorant pretender, attempting to speak of philosophy, and is obliged, as it is probably his first effort, to begin with the letter *a:* how far he will be able to proceed with the philosophical alphabet, I confess I am not able to determine.

In the next place I observe, that this *liberal* Reviewer has taken the unwarrantable liberty of twice reversing the order of my academical honours, which honours were not purchased by money, or undue influence, but by regular studies, examinations, and disputations, as is well known in Berlin and the University of Frankfort, and is testified by theses and diploma. I must repeat my enquiry, what was his motive?

This *learned* Reviewer appears to be a very ambiguous kind of being: on the one hand, he seems to belong to a kind of præternatural animals, which are not at all magnetic; and, on the other hand, one would suppose he secretly uses animal magnetism, and is fearful that too much light should be thrown on the subject.

My *learned friend* next observes, " indeed, had it not been for the title page, we could not have discovered what was the nature of the subject under discussion." And pray, my *learned friend*, do you now know the subject of my Treatise? Are you yet acquainted with it? To prove that you were not, when you sent your sarcastic nonsense to the publisher of the London Medical and Physical Journal, I shall make some remarks upon your manner of treating the subject of my little book, and on the *accuracy* of your quotations.

As to this writer's mode of making extracts, I would just observe, that no reader is able to judge properly of the merits of a work by reading a few unconnected passages, particularly when they are selected agreeably to the fine taste of my *learned friend;* and, as to his acknowledgment of his ignorance of the subject, I verily believe he has told the truth; for I am firmly persuaded, that, to this hour, he knows nothing about it.

My *very learned* friend next makes a very short extract " *on identity.*" When writing this part of my Treatise, I knew there were brains so dull, and skulls so thick, that some persons would not understand it, and this *very sensible* Reviewer has proved, that my conjectures were not altogether groundless.

The next passage he quotes is that on *Life and Death*, and he appears to be discontented with the idea, that no absolute death can exist; but it is not to be wondered at that he cannot separate himself from the idea of *annihilation*, for, from his illiberal manner of treating the subject, he causes us to suspect that he took up his pen to review my Treatise with the determination of annihilating the whole, if he could, even before he had thoroughly read it; and, to prove this, I need only refer that part of his review now under consideration, in which he takes the unwarrantable liberty of substituting the word *affected* for *effected**. As it is possible our *learned* Reviewer may not be sufficiently acquainted with the Latin language to know the difference between *afficere* and *efficere*, I shall refer him to the two English words in Dr. Johnson's Dictionary. Or, must I conclude, that my *very liberal* antagonist has wilfully altered the word to answer his own malicious purposes? I am not so illiberal as to affirm this, but until he explains himself on this head, I must conclude that he is extremely *ignorant.*

This *sagacious Critic* next observes, " Having waded through numberless, unintelligible paragraphs on life and death, we come to the following summing-up, the meaning of which we leave as a task for the ingenuity of our readers to discover," &c. This is the only part of the whole review which is logically correct; for, if he himself was unable to understand any part of the work, it was very natural that he should leave others to judge of its merits or demerits: if he continue to write in this way, it is possible he may in time learn to think and write correctly as well as others.

As to the two instances which I introduce in my Treatise to prove, that, in cases of concussion of the brain, neither the vegetative, nor the animal, but only the intellectual life was injured, this *candid* Reviewer has been pleased to give them the singular title of *stories;* and, moreover, has the audacity to tell his readers, that these " *amusing stories*," as he terms them, are inserted in order to shew " *the difference between natural and morbid sleep.*" From this remark it is evident, that this *clever*, this *diligent* Critic, when reviewing my book, did little more than cast his eye upon the title of each chapter, and perhaps upon one or two of the notes, for he makes a remark upon the note at the bottom of the page where this passage occurs, in which I inform my readers, that I do not exactly recollect whether I have heard these instances, or read them. A man, who is so wanting in exactness him-

* Obviously a typographical error.—EDITORS.

self, as this Reviewer evidently is, cannot, it appears, suffer exactness in another. But to *misrepresent* an author is the most serious offence of which a Reviewer can be guilty, and of this very serious offence I am compelled to charge this anonymous Critic. I now, therefore, address myself to this man, who would thus stab me, as it were, in the dark; and ask him, by what authority he has asserted, that I have adduced the two instances above alluded to, in order to shew "*the difference between natural and morbid sleep?*" In what part of my Treatise will he find such absurd nonsense?*

This *indefatigable* Reviewer at once skips over twenty pages, and then says, "our author proceeds to tell us, that some persons," &c. Why did he not affix the number of the page to each of the extracts, and also candidly inform his readers how many pages of my Treatise he did not read? But, to be again serious, I must notice a third misrepresentation which this *liberal* Reviewer has given of my meaning—he represents me as saying, that "some persons, who have been magnetised, become possessed of the faculty of *clairevoyance*, or what the Scotch term *second sight*." I have said no such thing. The only passage to which this misrepresentation can refer reads thus—"The degree which is called *clairevoyance* is nothing else than that faculty by which the natural and appropriate organs of sense do not exercise their functions," &c.; and, in a note at the bottom of the page, referring to the word *clairevoyance*, I remark, "this term has no correspondent English word; the nearest to it is what the Scotch term *second sight*." I could wish that this *perspicuous* Reviewer had not only a second and third sight, but many sights, and yet I am sure he will never have one *insight*, nor will he ever arrive at this state of *clairevoyance*. As a German, as the Reviewer is pleased to call me, and I am proud of the name, I cannot refrain quoting the sense of a passage from a poem of the celebrated German writer Schiller:

"Dem Ewig-blinden strahlt die Fackel nicht, sie kann nur zünden."

I will say nothing of the next two sentences, the former commencing thus, "In the *concluding* part," &c., and the latter thus, "He *concludes* with informing us," &c.

The Reviewer next observes, "His description of a person magnetised is the following—quietude and cheerfulness," &c. It appears that this *diligent* and *exact* Reviewer did not read my Preface. If he had, surely he would not have forgotten that I there say, "In the Appendix I propose to point out at large the particular degrees and varieties of the appearances and symptoms." This I have done; but this *impartial* Reviewer has quoted two short paragraphs from the fourth page of the Appendix, containing no more than an eighth part of the symptoms described under the second degree of magnetical phenomena, omitting altogether my description of the symptoms in the lowest degree, as well as those in the highest degree, and even without intimating that he has made this quotation from the Appendix. Is it possible to avoid suspecting, that this *very careful* Reviewer acted thus from no other motive than to make a medley of my book, or to represent it as a confused, unconnected heap of nonsense to persons who have not the book in their possession? This is a high degree of impudence indeed; but

"*Quo quis indoctior, eo impudentior?*"

I leave my *very learned* Reviewer to explain what he means by saying, "we have neither *limits*, nor inclination, to dwell longer," &c. Probably he meant to say, we have neither *space*, nor inclination, &c. but he himself will

---

* Our correspondent referred us to paragraph No. 60, which is very short, is entitled as mentioned above, and contains these stories; but we confess that we understand so little of the author's meaning throughout, that we cannot determine between the parties.—Editors.

perhaps favour his readers with an explanation of this new mode of concluding an *elaborate* criticism. If he meant to say that his own folly has no limits, I am perfectly satisfied with his use of the term, and would enquire, as Job did,

"*Shall vain words have an end?*"—Job xvi. 3.

As to the expression, "*German enthusiasm and folly,*" I remark, that I am not surprised to find in the production of this *able* Reviewer such a contradiction in terms as "*enthusiasm and folly:* this *logical* Critic perhaps does not know, that *no fool* can be an *enthusiast;* but, being sufficiently acquainted with his illogical nonsense throughout his review, I would only observe, that I am unable to reply to the term *German,* which he has prefixed to the above phrase, and by which he attacks the nation to which I belong. How can I reply to such scurrility? Shall I be so mean as to assert, that, because he is illiberal, the whole nation to which he belongs is illiberal too? By no means. I wish rather to refer my readers, and especially my *forgetful* Reviewer, to what I have said in the eighth page of my Preface; at the same time, however, I cannot restrain myself from uttering my suspicion, that, on account of his excessive illiberality, my Reviewer is not an Englishman, and, even if he were born in England, he surely cannot be of English extraction, for, when I consider his malicious illiberality, I am totally at a loss to conceive to what nation we can trace his origin, unless it be to that of the Caffres or Cannibals.

My *wise* Reviewer next observes, "indeed we might not have noticed it at all," &c. He would have given us one mark of wisdom had he been altogether silent on the subject, for the wisest man that ever lived said, "Even a fool, when he holdeth his peace, is counted wise."—Prov. xvii. 28. But by way of excuse for not having been totally silent, this *wise* man assigns as his motive for writing, "the exhortation of the author to professional men to clear the subject from all impurities;" but I addressed myself to *learned* professional men, (page 108,) and I apprehend my Reviewer belongs not to that class. He seems, therefore, to be an intruder, and, of his own accord, to have taken upon himself to represent the whole of the medical faculty. If he had this right, I should then be obliged to exclaim, "*O tempora! O mores!*" But I am far, very far, from conceiving, that the very respectable body of medical gentlemen in this country, a country which can boast of a Freind, a Mead, a Hunter, a Cullen, a Monro, a Cheselden, and others whom I could name, were I not fearful of affecting the modesty of those great men who are now living; I repeat, that I am far from thinking they would be willing to be represented by such an *illogical, illiberal* man.

I now dismiss this *criticus criticorum,* assuring him, and all others who review in such a manner as he does, that I shall act according to the advice of Solomon, "Answer not a fool"—Prov. xxvi. 4.; and shall address myself to those respectable Reviewers, who are guided solely by a love of truth. To such Reviewers I address myself in the words of my Preface, "incomplete and defective as this Treatise may be, yet the hope consoles me, that it may possibly prove a guide to others, endowed with more learning and greater talent than myself, to walk in the same path with better success: if, however, it should prove to be altogether deficient, I hope, that amidst the demolition of the system here laid down, materials may yet be found, which may be of service in the erection of a new and firmer edifice."

"I am open to conviction in any case where I may receive instruction: it is but a human mind that conceives a system, and a human mind that receives it."

M. LOEWE, M. D.

*London, November,* 1822.

